# Photosynthetic parameters and stomatal conductance in attached and detached balsam fir foliage

**DOI:** 10.1002/pei3.10059

**Published:** 2021-08-05

**Authors:** Matthew E. Akalusi, Fan‐Rui Meng, Charles P.‐A. Bourque

**Affiliations:** ^1^ Faculty of Forestry and Environmental Management University of New Brunswick Fredericton NB Canada

**Keywords:** balsam fir physiology, carbon dioxide, detached foliage, photosynthetic response, stomatal conductance

## Abstract

Leaf level gas‐exchange measurements can be made on detached foliage to address the challenge of access to the crown of tall trees. However, detachment may impact leaf gas exchange. This necessitates the study of gas‐exchange characteristics of foliage on detached branches to assess the feasibility of using detached branches for gas‐exchange analysis. We compared photosynthetic parameters and stomatal conductance in foliage of attached and detached branches of balsam fir [*Abies balsamea* (L.) Mill.] during the growing season. Data were analyzed using a linear mixed‐effect model, with fixed and random effects (branch status and measurement month, and tree number, respectively). Branch detachment had no significant effects on: (i) photosynthesis at the current ambient CO_2_ concentration (400 µmol mol^−1^, *A*_400_); (ii) maximum rates of Ribulose‐1,5‐bisphosphate (RuBP) carboxylation (*V*_cmax_) and regeneration (*J*_max_); (iii) the ratio of *J*_max_ to *V*_cmax_ (i.e., *J*_max_:*V*_cmax_), and (iv) stomatal conductance (*g*_s_) during the study period (*p* = 0.120–0.335). There was a strong seasonal effect on all gas‐exchange variables (*p* ≤ 0.001–0.015). Gas‐exchange measurements made on detached foliage during the warm summer months should be performed with care. Reliable gas‐exchange measurements can be obtained using balsam fir foliage on detached branches 50–80 cm in length, in cooler growing‐season months, up to 30 min after detachment.

## 1 INTRODUCTION

Gas‐exchange measurements benefit studies in tree physiology, facilitating the understanding of tree response to changes in environmental conditions. *In situ* gas‐exchange measurements, with the foliage attached to the branch are readily done in seedlings or small saplings because of ease‐of‐access. However, access to foliage becomes difficult when trees grow past the sapling stage (Gauthier & Jacobs, 2018). Experiments could be done with ladders, scaffolding, towers, etc. to reach foliage in the crown, several tens of meters above the ground. Choice of means of access to the tree crown is informed by the safety of researchers, effort and associated logistics required, flexibility of the system, and cost, etc. (McCarthy, 1988). Difficulties associated with canopy access would limit the number of measurements, many of which are required, in view of within‐tree crown and between‐tree variation. A trade‐off option in conducting gas‐exchange experiments could be accomplished by detaching foliage from tall trees and conveniently taking measurements on the ground (Meng & Arp, 1993; Richardson & Berlyn, 2002).

The process of detaching foliage from trees, that is, branch cutting, the interval between branch cutting and gas‐exchange measurements, or both, can affect the variables being assessed (Richardson & Berlyn, 2002). The photosynthetic capacity of cut foliage may be affected by resultant changes in cell turgor or stomatal aperture (Clark, 1954; Lundegardh, 1931), and drawdown of moisture which may occur because of continued transpiration (Meng & Arp, 1993). However, detachment is still a viable method for measuring gas exchange (see researchers listed in Table 1), but few studies have reported on its effects on gas‐exchange measurements (Clark, 1954; Gauthier & Jacobs, 2018; Koike & Sakagami, 1984; Meng & Arp, 1993).

**TABLE 1:** Studies reporting *V*_cmax_ and *J*_max_ from detached foliage

Source	Species studied
Niinemets et al. (1998)	*Populus tremula* L., *Fraxinus excelsior* L., *Tilia cordata* Mill., *Corylus avellana* L.
Medlyn et al. (2002)	*Pinus pinstar* Aiton.
Warren et al. (2003)	*Pinus sylvestris* L.
Ethier et al. (2006)	*Pseudotsuga menziesii* (Mirb.) Franco
Goodine et al. (2008)	*Abies balsamea* (L.) Mill.
Wang et al. (2008)^a^	*Fagus crenata* Blume
Merilo et al. (2009)	*Picea abies* L.
Woodruff et al. (2009)	*Pseudotsuga menziesii*
Drake et al. (2010)	*Pinus taeda* L.
Whitehead et al. (2011)^a^	*Nothofagus solandrii* (Hook.f.) Oerst.
Raim et al. (2012)	*Picea abies*
Katahata et al. (2014)	*Daphniphyllum humile* Maxim

^a^ Studies that only report *V*_cmax_.

Clark (1954), Koike and Sakagami (1984), Meng and Arp (1993), and Gauthier and Jacobs (2018) studied the effect of detachment on gas exchange in Norway and red spruce [*Picea abies* (L.) Karst., *P*. *rubens* Sarg.], species of birch (i.e., *Betula ermanii* Cham., *B*. *platyphylla* Sukatchev. and *B*. *maximowicziana* Sukatchev), black walnut (*Juglans nigra* L.), red and white oak (*Quercus rubra* L. and *Q*. *alba* L.) and found that the period within which gas‐exchange rates remained unchanged ranged from 3 to 20 min in foliage of differing sizes.

Gas‐exchange studies available in the literature have generally assessed the impacts of detachment on the rate of photosynthesis (*A*) in tree foliage, and even fewer, on stomatal conductance (*g*_s_). This study reports on the impacts of detachment on photosynthetic parameters, including maximum rate of Ribulose‐1,5‐bisphosphate (RuBP) carboxylation (*V*_cmax_; μmol m^−2^ s^−1^) and the maximum rate of RuBP regeneration (*J*_max_; μmol m^−2^ s^−1^), derived from measurements of *A* (μmol m^−2^ s^−1^) relative to intercellular carbon dioxide (*C*_i_; μmol mol^−1^), in response to changing levels of carbon dioxide (alias CO_2_ response or *A*‐*C*_i_ curves). This is important because the Farquhar et al. (1980) model is the most widely used in the analysis of CO_2_‐photosynthesis response in trees. Two key parameters in the model, that is, *V*_cmax_ and *J*_max_, have been reported on, in several *in situ* gas‐exchange studies (e.gDiaz‐Espejo et al., 2006; Fujita et al., 2012; Wilson et al., 2000; Xu & Baldocchi, 2003). Several researchers have reported on these parameters derived from measurements directly obtained from detached foliage (Table 1). Studies by Wang et al. (2008) and Whitehead et al. (2011) only reported *V*_cmax_.

Warren et al. (2003), Ethier et al. (2006), Goodine et al. (2008), Merilo et al. (2009), Woodruff et al. (2009), Drake et al. (2010), and Katahata et al. (2014) referred to preliminary measurements conducted in attached and detached foliage to compare gas‐exchange rates prior to conducting measurements on which their studies were based. The study by Drake et al. (2010) is the only one we are aware of that has reported on the effects of foliage detachment on *V*_cmax_ and *J*_max_. To our knowledge, this is the first study that assesses *V*_cmax_ and *J*_max_ of attached and detached foliage in balsam fir, and the response of these parameters in such foliage to seasonal variation. The objective of this study was to report field observations of the parameters of photosynthesis and stomatal conductance before and after branch detachment during early summer‐to‐fall conditions.

## 2 MATERIALS AND METHODS

### 2.1 Study site

The study was conducted at the University of New Brunswick Woodlot (45° 56ʹ N, 66° 40ʹ W), New Brunswick, Canada, which lies within the Maritime Lowlands Ecoregion of the Atlantic Maritime Ecozone (Ecological Stratification Working Group, 1996). Humo‐ferric podzols and gray luvisols are the dominant soil types in the Ecoregion, with significant areas of gleysols, fibrisols, and mesisols (Ecological Stratification Working Group, 1996). The soils are well‐drained, sandy‐clay loams, from the soil surface to a depth of 30 cm with <20% coarse fragments. The depth to the compacted layer ranges from <30 to 30–65 cm (Ecological Stratification Working Group, 1996; Canadian Soil Information Service, 2019).

### 2.2 Gas‐exchange measurements

Nine balsam fir trees, ranging in age from 15 to 20 years, with diameter at breast heights (DBH) between 9 and 19 cm, were selected. Photosynthesis measurements were made in July, August, October, and November of 2019 with respective mean monthly temperature and total precipitation as follows: 20℃ and 70.7 mm; 18.2℃ and 79.9 mm; 8.2℃ and 105.1 mm; −0.6℃ and 118.1 mm (after Environment & Climate Change Canada, 2019).

The development of CO_2_ response curves and measurement of stomatal conductance commenced each day at 09:00 h (Local Atlantic Daylight Time) using a CIRAS‐2 Photosynthesis System (PP Systems). Measurements were made, first on 1‐year‐old foliage which were totally enclosed in the conifer chamber (cuvette), while branches were attached to each tree, then again on the same foliage, following detachment, each detached branch was around 50‐ to 80‐cm long. Measurements were made under saturating light, with photosynthetically active radiation (PAR) ranging from 1190 to 1200 μmol m^−2^ s^−1^ supplied by a tungsten halogen light unit (PP Systems). Reference CO_2_ concentration (*C*_ref_) was changed in descending steps of 400, 200, 100, and 50, and ascending steps of 400, 800, 1,000, 1,400, and 1,800 μmol mol^−1^. Flow rate was set at 400 ml min^−1^, chamber temperature at 18℃, and relative humidity at 70%. Chamber temperature was set at 18℃ because conifers are known to undergo optimal photosynthetic activity at temperatures lower than 25℃ (Lin et al., 2012; Wieser et al., 2010), the reference temperature on which the Farquhar et al., (1980) model is based. Before starting each response curve, the foliage was acclimated to cuvette conditions for 3 min. Two minutes were allowed for stabilization of *A* after each change in CO_2_ concentration before the variable was measured. Stomatal conductance (mmol m^−2^ s^−1^) was measured simultaneously. Each CO_2_ response curve was completed within 30 min. Fifty‐six response curves were developed over the study period.

On completion of the response curves for both attached and detached foliage, all needles on each foliage (*n* = 30–50) were removed, and projected leaf area (½ of total leaf area) was determined by scanning the needles with a CanoScanLiDE 110 Flatbed Scanner (Canon Canada Inc). The image was then analyzed with Image J software (National Institutes of Health). Projected leaf area was subsequently used to calculate *A* on a leaf‐area basis.

### 2.3 Data analysis

The Farquhar et al., (1980) photosynthesis model is comprised of two parts. The first part is

(1) $$A_{c}=\frac{V_{c\max}C_{i}‐\Gamma^{*}}{C+K_{c}\left(1+\frac{O_{i}}{K_{o}}\right)}‐R_{d},$$

where *A*_c_ is the Ribulose‐1,5‐bisphosphatecarboxylase/oxygenase (Rubisco)‐limited net photosynthesis (μmol m^−2^ s^−1^), *K*_c_ and *K*_o_ the Michaelis–Menten constants for carboxylation and oxygenation (404 μmol mol^−1^ and 248 mmol mol^−1^, respectively; von Caemmerer et al., 1994), *O*_i_ the oxygen concentration at the site of carboxylation (210 mmol mol^−1^), and *R*_d_ dark respiration (μmol m^−2^ s^−1^). The second part is as follows:

(2) $$A_{r}=\frac{J_{\max}C_{i}‐\Gamma^{*}}{4C_{i}+8\Gamma^{*}}‐R_{d},$$

where *A*_r_ is the Ribulose‐1,5‐bisphosphate (RuBP)‐limited net photosynthesis (μmol m^−2^ s^−1^), *C*_i_ the intercellular CO_2_ concentration at the current ambient CO_2_ concentration, ~400 μmol mol^−1^, and Г^*^ the CO_2_ compensation point in the absence of dark respiration (μmol mol^−1^), at which point there is no net assimilation. The CO_2_ compensation point in the absence of dark respiration is determined by the following equation:

(3) $$\Gamma^{*}=\frac{K_{c}O_{i}k_{o}}{2K_{o}k_{c}},$$

where *k*_c_ and *k*_o_ are the turnover rates for RuBP carboxylase and RuBP oxygenase (2.5s^−1^ and 0.55 s^−1^ or 0.22*k*_c_; after Farquhar et al., 1980).

The key parameters *V*_cmax_ and *J*_max_ in eqn.’s 1 and 2 were estimated from regression analysis of *A*‐*C*_i_ curves (Wullschleger, 1993), which necessitates the designation *a priori* of a *C*_i_‐threshold, at which there is a switch between RuBP‐saturated and limited portions of the curve (Manter & Kerrigan, 2004). Generally, it is assumed that at low *C*_i_, *A* is solely limited by *V*_cmax_, whereas at high *C*_i_, *A* is limited by *J*_max_. The parameters *V*_cmax_ and *J*_max_ were estimated using the lower section of the response curve, when *C*_i_ was approximately ≤300 µmol mol^−1^, and the entire curve, respectively (Farquhar et al., 1980; Wullschleger, 1993; Xu & Baldocchi, 2003), that is,

(4) $$\text{CE}=\frac{V_{c\max}}{\Gamma^{*}+K_{c}\left(1+\frac{O_{i}}{K_{o}}\right)},$$

(5) $$V_{c}=\frac{V_{c\max}C_{i}}{C_{i}+K_{c}\left(1+\frac{O_{i}}{K_{o}}\right)},\;\text{and}$$

(6) $$J_{\max}=V_{c}\left(4+\frac{8\Gamma^{*}}{C_{i}}\right),$$

after Farquhar et al., 1980 and von Caemmerer, 2000), where *CE* and *V*_c_ in eqn.’s 4 and 5 are the carboxylation efficiency, representing the initial slope of the CO_2_ response curve and the rate of carboxylation, respectively. The equation constants, that is, *K*_c_, *K*_o_, and Г^*^, having been derived at an ambient temperature of 25℃ were converted to values at the reference temperature (i.e., 18℃) with the following equations:

(7) $$K_{c}\left(T\right)=K_{c}\left(25^{\circ }C\right)\exp\left(59356\left(\frac{0.000404\left(T‐25\right)}{T+273}\right)\right),$$

(8) $$K_{o}\left(T\right)=K_{o}\left(25^{\circ }C\right)\exp\left(35948\left(\frac{0.000404\left(T‐25\right)}{T+273}\right)\right),\;\text{and}$$

(9) $$\Gamma^{*}\left(T\right)=\Gamma^{*}\left(25^{\circ }C\right)+0.188\left(T‐25\right)+0.0036{\left(T‐25\right)}^{2}$$

Lambers et al., (1998), where *T* is the reference temperature at which the CO_2_ response curve was developed.

The linear mixed‐effects model option in SPSS Statistical software (*ver*. 24.0, IBM Corp.) was used to analyze the maximum rate of photosynthesis at the current ambient CO_2_ concentration (i.e., photosynthesis at 400 µmol mol^−1^, *A*_400_), *V*_cmax_, *J*_max_, ratio of *J*_max_ to *V*_cmax_ (i.e., *J*_max_:*V*_cmax_), and mean *g*_s_. Branch status (attached or detached foliage) and measurement month (July, August, October, and November) were the fixed factors, and tree number was the random factor. This analysis was done as a result of the non‐independence of measurements made on both foliage types from each tree, and repeated sampling during the study period.

The model equations used are as follows,

(10) $$\begin{array}{cc} Y & =\beta_{0}+\beta_{1}\;\text{branch status}+\beta_{2}\;\text{measurement month}\\ & \;+b_{1}\;\text{tree number}+\varepsilon \end{array}$$

(11) $$\begin{array}{cc} Y & =\beta_{0}+\beta_{1}\;\text{branch status}+\beta_{2}\;\text{month of measurement}\\ & \;+\beta_{3}\;\text{branch status}\times \text{month of measurement}\\ & \;+b_{1}\;\text{tree number}+\varepsilon \end{array}$$

In eqn.’s 10 and 11, *Y* is the gas‐exchange parameter of interest, *β_0_*, *β_1_*, *β_2_*, *β*_3_, and *b*_1_ are regression coefficients to be estimated, and ε is the regression error term.

## 3 RESULTS

**FIGURE 1:**
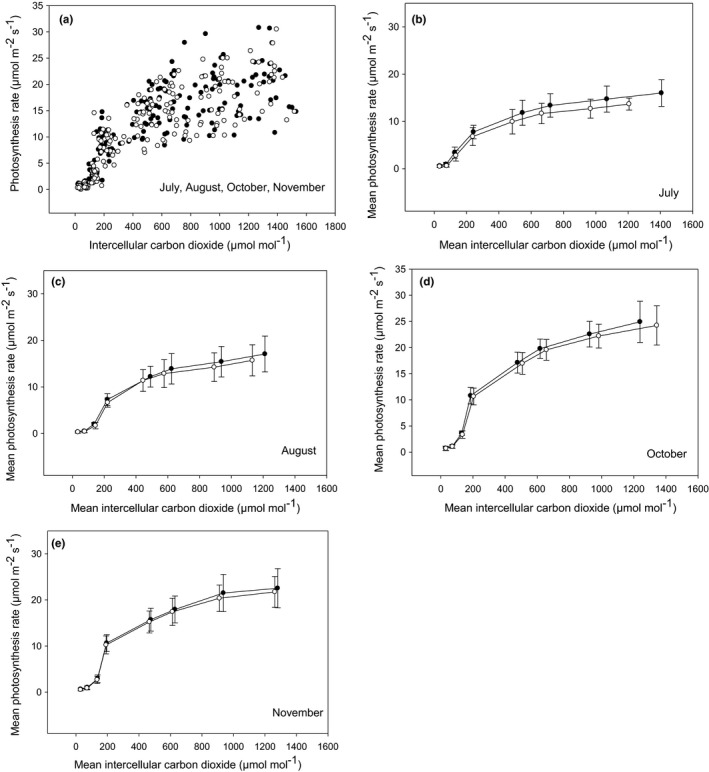
The response of maximum photosynthesis (*A*; µmol m^−2^ s^−1^) to intercellular CO_2_ (*C*_i_; µmol mol^−1^) of attached (closed circles) and detached balsam fir foliage (open circles) assessed in July through to November 2019 (a). The mean monthly response ±the standard deviation (error bars; panels b–e

**FIGURE 2:**
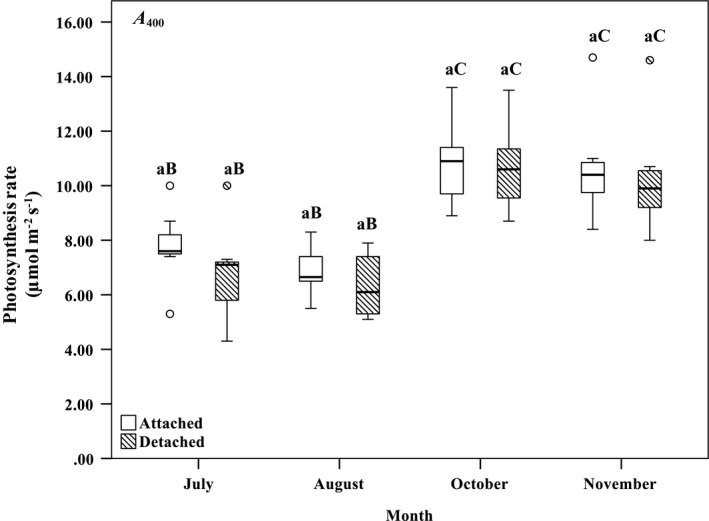
Box plots of rate of photosynthesis at 400 µmol mol^−1^ (*A*_400_; µmol m^−2^ s^−1^) of attached and detached balsam fir foliage assessed in July through to November 2019. The thick horizontal lines in the boxes represent the median of plotted values. The bottom and top edges of the boxes denote the 25^th^ and 75^th^ percentiles, whereas the ends of the whiskers correspond to the 10^th^ and 90^th^ percentiles. Similar lowercase letters indicate no significant difference regarding branch status (*p* > 0.05). Differences in uppercase letters indicate significant differences regarding measurement month (*p* < 0.05). Comparisons were made in relation to reference values for branch status and measurement month

**FIGURE 3:**
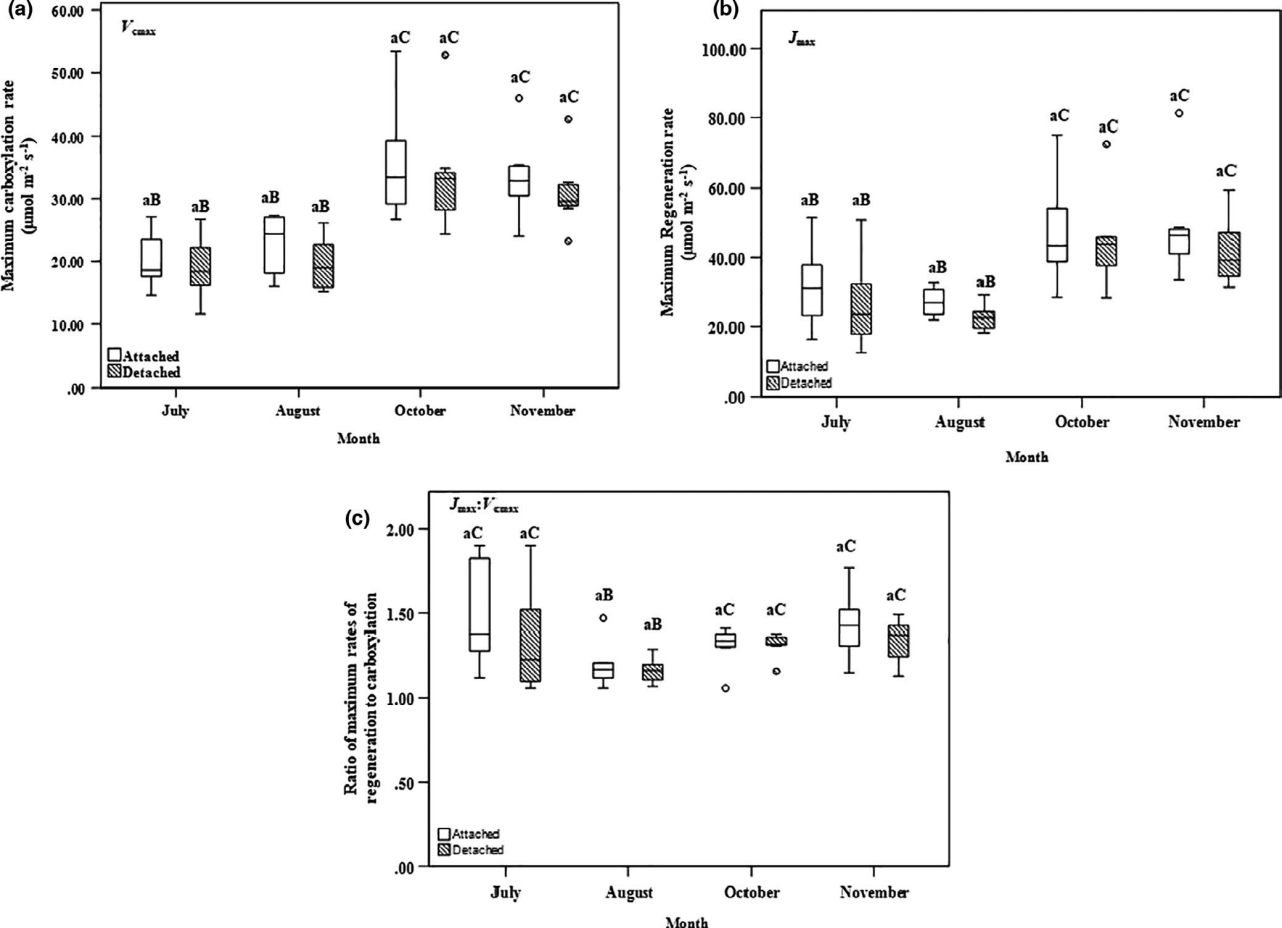
Box plots of (a) the maximum rate of ribulose‐1,5‐bisphosphate carboxylation (*V*_cmax_; µmol m^−2^ s^−1^), (b) the maximum rate of ribulose‐1,5‐bisphosphate regeneration (*J*_max_; µmol m^−2^ s^−1^), and (c) the ratio of the maximum rates of ribulose‐1,5‐bisphosphate regeneration (*J*_max_;µmol m^−2^ s^−1^) to carboxylation (*V*_cmax_;µmol m^−2^ s^−1^) of attached and detached balsam fir foliage assessed in July through to November 2019. See Figure 2 for box‐plot descriptions. Similar lowercase letters above box plots indicate no significant difference regarding branch status (*p* > 0.05). Differences in uppercase letters above box plots indicate significant differences regarding measurement month (*p* < 0.05). Comparisons were made in relation to reference values for branch status and measurement month

**FIGURE 4:**
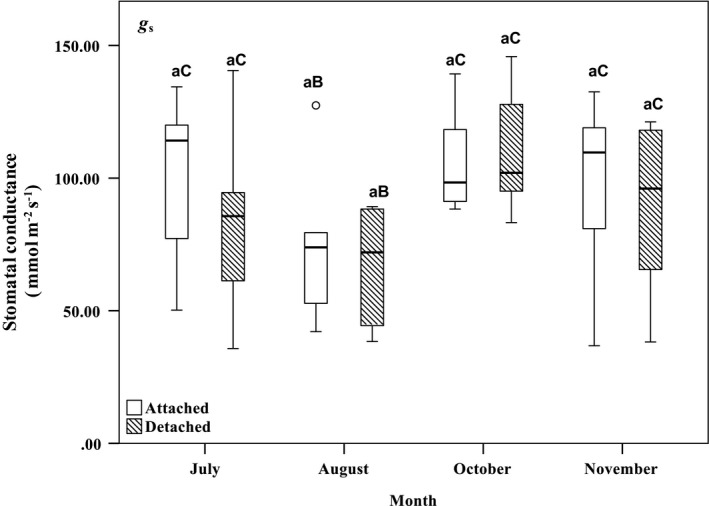
Box plots of mean stomatal conductance (*g*_s_; mmol m^−2^ s^−1^) of attached and detached balsam fir foliage assessed in July through to November 2019, varying *C*_ref_ from 50 to 1800 µmol mol^−1^. The thick horizontal lines in the boxes represent the median of plotted values. See Figure 2 for box‐plot descriptions. Similar lowercase letters indicate no significant difference regarding branch status (*p* > 0.05). Differences in uppercase letters indicate significant differences regarding measurement month (*p* < 0.05). Comparisons were made in relation to reference values for branch status and measurement month

As shown in Figure 1a–e, A increased with varying *C*_ref_ (from 50 to 1800 µmol mol^−1^) and ranged from 0.00 to 30.79 µmol m^−2^ s^−1^ and from 0.00 to 30.50 µmol m^−2^ s^−1^ in attached and detached foliage, respectively. The parameter *A*_400_ ranged from 5.30 to 14.70 µmol m^−2^ s^−1^ and from 4.30 to 14.60 µmol m^−2^ s^−1^ in attached and detached foliage, respectively. The highest mean *A*_400_ values for attached and detached foliage were 10.80 ± 1.56 and 10.66 ± 1.61 µmol m^−2^ s^−1^, respectively, and were found to occur in October. The range for *V*_cmax_ in attached and detached foliage, was 14.58–53.36 and 11.62–52.78 µmol m^−2^ s^−1^, respectively. The highest mean *V*_cmax_ in attached and detached foliage was 35.79 ± 9.44 and 33.59 ± 9.29 µmol m^−2^ s^−1^, respectively, also occurred in October. The range for *J*_max_ in attached and detached foliage, was 16.28–81.33 and 12.44–72.71 µmol m^−2^ s^−1^. The mean *J*_max_ value in November of 48.20 ± 14.37 µmol m^−2^ s^−1^ was the highest in attached foliage. In detached foliage, this occurred in October, with a value of 44.49 ± 13.98 µmol m^−2^ s^−1^. The values of *J*_max_:*V*_cmax_ in attached and detached foliage ranged from 1.06 to 1.90 (non‐dimensional). The highest mean *J*_max_:*V*_cmax_ were 1.51 ± 0.33 and 1.35 ± 0.12 for attached and detached foliage, and occurred in July. Stomatal conductance (*g*_s_) in both attached and detached foliage ranged from 20 to 150 mmol m^−2^ s^−1^. Some exceptions between 150 and 230 mmol m^−2^ s^−1^ coincided with periods of active rainfall that increased foliage surface wetness and cuvette relative humidity, which led to higher‐than‐normal readings of *g*_s_. In attached foliage, mean *g*_s_ at the lowest *C*_ref_, 50 µmol mol^−1^ ranged from 74.49 to 117.24 mmol m^−2^ s^−1^, and at the highest *C*_ref_, 1800 µmol mol^−1^ it ranged from 67.71 to 106.5 mmol m^−2^ s^−1^. In detached foliage, mean *g*_s_ at 50 mol mol^−1^ ranged from 67.57 to 119.01 mmol m^−2^ s^−1^, and at 1800 µmol mol^−1^ it ranged from 60.20 to 117.13 mmol m^−2^ s^−1^. Across *C*_ref_ levels, mean *g*_s_ in attached and detached foliage samples ranged from 36.80 to 139.27 and from 35.74 to 145.79 mmol m^−2^ s^−1^, respectively. The highest mean *g*_s_ in attached and detached foliage samples, 106.42 ± 22.45 and 110.96 ± 24.09 mmol m^−2^ s^−1^, respectively, also occurred in October (Figures 2, 3, 4). The branch status (fixed effect) and tree number (random effect) did not have significant effects on *A*_400_, *V*_cmax_, *J*_max_, *J*_max_:*V*_cmax_, and mean *g*_s_ (*p* = 0.217, 0.181, 0.120, 0.164, 0.335, respectively). The month of measurement did have a statistically significant effect on *A*_400_, *V*_cmax_, and *J*_max_ (*p* < 0.001, respectively), but branch status ×month of measurement had no significant effect on the parameters (*p* = 0.916, 0.984, 0.980, respectively). Month of measurement only had a significant effect on *J*_max_:*V*_cmax_ and mean *g*_s_ when the main effects of the fixed factors were assessed (*p* = 0.013, 0.009, respectively), as the introduction of the interaction term, which was not significant (*p* = 0.688, 0.793, respectively) rendered the effect of month on *J*_max_:*V*_cmax_ and mean *g*_s_ statistically non‐significant. With the interaction between the fixed factors having no significant effect on all gas‐exchange parameters, the interaction term was ignored (Tables 2 and 3).

**TABLE 2:** Output of mixed‐effect model analysis (fixed effects; branch status and month) of *A*_400_, *V*_cmax_, *J*_max_, *J*_max_:*V*_cmax_, and *g*_s_ in attached and detached balsam fir foliage assessed in July through to November 2019

Parameter^a^	Estimate	Std. Error	*df*	*t*‐value	*p*‐value
*A*_400_					
Branch status					
Attached	0.525	0.418	42.571	1.254	0.217
Detached^b^	0	0	.	.	.
Month					
July	−3.222	0.577	47.417	−5.585	0.000
August	−3.447	0.604	48.842	−5.707	0.000
October	0.310	0.574	44.376	.539	0.593
November^b^	0	0	.	.	.
*V*_cmax_					
Branch status					
Attached	2.285	1.684	46.837	1.357	0.181
Detached^b^	0	0	.	.	.
Month					
July	−12.439	2.309	48.971	−5.388	0.000
August	−10.885	2.409	49.028	−4.518	0.000
October	2.547	2.307	47.840	1.104	0.275
November^b^	0	0	.	.	.
*J*_max_					
Branch status					
Attached	4.811	3.038	47.968	1.584	0.120
Detached^b^	0	0	.	.	.
Month					
July	−15.849	4.170	49.280	−3.800	<0.001
August	−19.942	4.352	49.322	−4.582	<0.001
October	1.286	4.165	48.604	0.309	0.759
November^b^	0	0	.	.	.
*J*_max_:*V*_cmax_					
Branch status					
Attached	0.074	0.052	45.300	1.415	0.164
Detached^b^	0	0	.	.	.
Month					
July	0.466	0.072	48.500	0.647	0.520
August	−0.205	0.075	48.930	−2.729	0.009
October	−0.071	0.072	46.580	−0.994	0.325
November^b^	0	0	.	.	.
*g*_s_					
Branch status					
Attached	7.240	7.435949	47.159	.974	0.335
Detached^b^	0	0	.	.	.
Month					
July	−4.216	10.274	49.189	−0.410	0.683
August	−25.650	10.897	50.262	−2.354	0.023
October	14.272	10.206	47.800	1.398	0.168
November^b^	0	0	.	.	.

^a^ *A*_400_ = rate of photosynthesis at 400 µmol mol^−1^; *V*_cmax_ = maximum rate of ribulose‐1,5‐bisphosphate carboxylation; *J*_max_ = maximum rate of ribulose‐1,5‐bisphosphate regeneration; *J*_max_:*V*_cmax_ = ratio of the maximum rates of ribulose‐1,5‐bisphosphate regeneration to carboxylation; *g*_s_ = stomatal conductance; *df* = degrees of freedom.

^b^ Reference set to zero.

**TABLE 3:** Output of mixed‐effect model analysis (random effect; tree number) of *A*_400_, *V*_cmax_, *J*_max_, *J*_max_:*V*_cmax_, and *g*_s_ in attached and detached balsam fir foliage samples assessed in July through to November 2019

Parameter	Estimate	Std. Error	Wald Z	*p*‐value
*A*_400_	0.090	0.283	0.319	0.749
*V*_cmax_	0.244	2.414	0.101	0.919
*J*_max_	2.933	7.884	0.372	0.710
*J*_max_:*V*_cmax_	0.002	0.003	0.449	0.654
*g*_s_	108.231	325.566	0.332	0.740

See Table 2 for parameter definitions.

## 4 DISCUSSION

The results of this study showed that gas exchange in detached foliage did not differ significantly from those in attached foliage 30 min after detachment (*p* = 0.120–0.335), the period required for completion of a single CO_2_ response curve. This may be because the water status of the detached foliage was not affected by detachment during the development of individual response curves. Hydraulically efficient stems ensure adequate water supply to the leaves such that the water loss through transpiration can be replaced readily, ensuring that deficits in leaf water supply are minimized, and stomata remain open for CO_2_ uptake (Bucci et al., 2019; Xiong et al., 2018). Photosynthesis has been documented among the primary physiological processes affected by water stress (Galmes et al., 2011). Detachment does not initially affect the rate of net photosynthesis and stomatal conductance of detached foliage, but over a prolonged period, cumulative moisture losses from leaves following detachment cause reductions. Nevertheless, a small and immediate increase in measurement variations occurs following twig detachment (Meng & Arp, 1993).

The opening and closing of stomata embedded in the epidermis simultaneously control plants’ water loss during transpiration and their uptake of CO_2_. Water loss through plant leaves during transpiration, which results in stomatal closure, occurs from an imbalance between water effluxes and influxes. During transpiration, water‐conducting elements contain water columns, which are under tension. Consequently, a water‐potential gradient between mesophyll tissue and xylem elements occurs, ensuring the movement of water against flow resistances (Buckley, 2005; Heber et al., 1986). Stomatal response to foliage detachment is a consequence of the positive relationship between stomatal aperture and turgor pressure of the guard cells, which form the pore, but it has a negative relationship with turgor pressure of adjacent epidermal cells, the more effective, of these two opposing pressures, in regulating aperture (Buckley, 2005).

The rate at which CO_2_ enters the leaf is regulated by resistances caused by the opening and closing of the stomata, and the internal pathways within the leaf mesophyll. The reduction in plant photosynthesis arising from moisture stress is generally explained by an increase in the resistance to the movement of CO_2_ through the stomata and mesophyll pathways to the site of fixation in the chloroplast. These resistances are substantially increased under such conditions because of low leaf water potentials (Beadle et al., 1973; Brix, 1962; Crafts, 1968; Puritch, 1973; Slatyer, 1967).

Generally, an increase in water stress results in a two‐phase photosynthetic response, with a xylem water potential threshold, above which little or no change in photosynthesis occurs, and below which it rapidly decreases. This two‐phase response varies among species (Melzack et al., 1985) and has been observed in conifers in studies of the effects of water stress in *Pinus taeda* L., (Brix, 1962) four species of the genus *Abies* [i.e., *A*. *balsamea*, *A*. *amabilis* (Doug) ex. Loud., *A*. *Lasiocarpa* (Hook.) Nutt., and *A*. *Grandis* (Doug) Lindl.; Puritch, 1973], and *Picea sitchensis* (Bong.) Carr. (Watts & Neilson, 1978). Brix (1962) found that photosynthesis in *P*. *taeda*, under water stress was stable until it increased to 405 kPa, following which there was a decline. Puritch (1973) reported similar stability of photosynthesis under water stress in the four species of the genus *Abies* studied, up until stresses between 900 and 1,100 kPa followed by a decline. Watts and Neilson (1978) observed stable photosynthesis in *P*. *sitchensis* under water stress up until 1500 kPa, after which there was a decline. The results of this study may, therefore, be an indication that the period during which a CO_2_ response curve was assessed for detached foliage, occurred at a xylem water potential that allowed photosynthesis to remain at rates that were largely like those in attached foliage samples.

The values of *A*_400_, *V*_cmax_, *J*_max_, and *J*_max_:*V*_cmax_ for attached and detached foliage all showed strong seasonal variation (*p* ≤ 0.001–0.013). The studies by Xu and Baldocchi (2003) and Wilson et al., (2000) showed seasonal variation in the gas‐exchange parameters measured, with peak values recorded for the gas‐exchange parameters measured in spring and summer, respectively, with declines in subsequent seasons. In this study, however, *A*_400_, *V*_cmax_, and *J*_max_ increased from summer to fall. This is attributable to the fact that Xu and Baldocchi (2003) worked on *Q*. *douglasii* Hook & Arn, and Wilson et al., (2000) worked on *Q*. *prinus* L., *Q*. *alba*, *Acer rubrum* L., *A*. *saccharum* Marsh, and *Nyssa sylvatica* Marsh, all deciduous species. Conifers and deciduous species have different photosynthetic responses to changing atmospheric temperature, with conifers (including balsam fir) attaining higher photosynthetic rates at lower temperatures compared to deciduous species (Lin et al., 2012), such as obtained during fall. The pattern of seasonal variation in *J*_max_:*V*_cmax_, in attached and detached samples showed that the highest value in both foliage types occurred in July. Though the pattern of seasonal variation in *J*_max_:*V*_cmax_ is a departure from those seen in *A*_400_, *V*_cmax_, and *J*_max_, the values ranged between 1 and 3, the range within which *J*_max_:*V*_cmax_ is reported to fluctuate, and reflects the balance between RuBP carboxylation and regeneration (Kattge & Knorr, 2007; Onoda et al., 2005; Robakowski et al., 2002; Walcroft et al., 1997; Wullschleger, 1993). Mean *g*_s_ across *C*_ref_‐levels showed a seasonal trend and peaked in October. A similar trend was reported by Beadle et al., (1985) in their study of *Pinus sylvestris* L. (another conifer). The seasonal effect on detached foliage also varied in relation to prevailing atmospheric conditions with a general trend of significantly lower gas‐exchange parameters in August compared to those in October and November. This is because of higher temperatures and accompanying greater vapor pressure deficit between foliage and the air in the summer, than in the fall (Berry & Bjorkman, 1980).

## 5 CONCLUSIONS

A comparison was made of photosynthetic parameters and stomatal conductance in foliage of attached and detached branches of balsam fir at different times during the growing season, using a linear mixed‐effect model. Branch detachment did not have a significant effect on gas‐exchange parameters studied, in particular *A*_400_, *V*_cmax_, *J*_max_, *J*_max_:*V*_cmax_, and *g*_s_. There was, however, a strong seasonal effect on the parameters. The trend of the data from this study indicates that the photosynthetic parameters and rate of photosynthesis were observed to be highest in October and November, an indication that the optimum temperature of balsam fir is low. The impact of detachment on *g*_s_ during July and August indicated that caution is required when gas‐exchange measurements are made over long durations, during warm summer months. We can conclude that detachment had a negligible impact on gas‐exchange measurements in balsam fir foliage when carried out on branches 50‐ to 80‐cm long, for up to 30 min. The results from balsam fir are more reliable when gas‐exchange measurements are done during cooler months.

## CONFLICT OF INTEREST

The authors declare that they have no conflict of interest.

## AUTHOR CONTRIBUTION

MEA, FRM, and CPAB conceived the ideas; MEA collected the data, designed the methods used, did the data analysis, and led the writing of the manuscript; FRM and CPAB reviewed the drafts, made amendments to the manuscript, and gave final approval for its publication.

## Data Availability

The data that support the findings of this study are openly available in figshare at https://doi.org/10.6084/m9.figshare.14791716.
